# A fibroblast-associated signature predicts prognosis and immunotherapy in esophageal squamous cell cancer

**DOI:** 10.3389/fimmu.2023.1199040

**Published:** 2023-05-29

**Authors:** Qianhe Ren, Pengpeng Zhang, Xiao Zhang, Yanlong Feng, Long Li, Haoran Lin, Yue Yu

**Affiliations:** ^1^ Department of Thoracic Surgery, The First Affiliated Hospital of Nanjing Medical University, Nanjing, China; ^2^ Department of Thoracic Surgery, Nanjing Gaochun People’s Hospital, Nanjing, China

**Keywords:** esophageal squamous cell carcinoma, fibroblasts, risk signature, tumor immune microenvironment, immunotherapy

## Abstract

**Background:**

Current paradigms of anti-tumor therapies are not qualified to evacuate the malignancy ascribing to cancer stroma’s functions in accelerating tumor relapse and therapeutic resistance. Cancer-associated fibroblasts (CAFs) has been identified significantly correlated with tumor progression and therapy resistance. Thus, we aimed to probe into the CAFs characteristics in esophageal squamous cancer (ESCC) and construct a risk signature based on CAFs to predict the prognosis of ESCC patients.

**Methods:**

The GEO database provided the single-cell RNA sequencing (scRNA-seq) data. The GEO and TCGA databases were used to obtain bulk RNA-seq data and microarray data of ESCC, respectively. CAF clusters were identified from the scRNA-seq data using the Seurat R package. CAF-related prognostic genes were subsequently identified using univariate Cox regression analysis. A risk signature based on CAF-related prognostic genes was constructed using Lasso regression. Then, a nomogram model based on clinicopathological characteristics and the risk signature was developed. Consensus clustering was conducted to explore the heterogeneity of ESCC. Finally, PCR was utilized to validate the functions that hub genes play on ESCC.

**Results:**

Six CAF clusters were identified in ESCC based on scRNA-seq data, three of which had prognostic associations. A total of 642 genes were found to be significantly correlated with CAF clusters from a pool of 17080 DEGs, and 9 genes were selected to generate a risk signature, which were mainly involved in 10 pathways such as NRF1, MYC, and TGF-Beta. The risk signature was significantly correlated with stromal and immune scores, as well as some immune cells. Multivariate analysis demonstrated that the risk signature was an independent prognostic factor for ESCC, and its potential in predicting immunotherapeutic outcomes was confirmed. A novel nomogram integrating the CAF-based risk signature and clinical stage was developed, which exhibited favorable predictability and reliability for ESCC prognosis prediction. The consensus clustering analysis further confirmed the heterogeneity of ESCC.

**Conclusion:**

The prognosis of ESCC can be effectively predicted by CAF-based risk signatures, and a comprehensive characterization of the CAF signature of ESCC may aid in interpreting the response of ESCC to immunotherapy and offer new strategies for cancer treatment.

## 1 Introduction

Esophageal cancer (EC) is a prevalent form of cancer, ranking eighth among all cancer types, and is also the sixth most common cause of cancer-related deaths globally (1). It primarily consists of two major subtypes: esophageal squamous cell cancer (ESCC) and esophageal adenocarcinoma (EAC). ESCC accounts for the majority of esophageal cancer cases worldwide with a higher incidence in East Asia and Africa. On the other hand, esophageal adenocarcinoma (EAC) is more prevalent in many developed countries (2). Despite the great achievements in the management of ESCC, including surgery, endoscopic resection, chemoradiotherapy, and immunotherapy, this aggressively malignant tumor still extremely threatens patients’ health attributed of its heterogeneity (3). Limited understanding of its molecular etiology further makes up for the poor prognosis (4). Thus, exploring the properties and identifying novel biomarkers for ESCC is urgently needed.

Targeted therapies have made remarkable strides in the management of diverse neoplastic conditions, such as esophageal squamous cell carcinoma (ESCC). Immunotherapy, encompassing the utilization of immune checkpoint inhibitors (ICIs)/immunomodulators, therapeutic vaccines, monoclonal antibodies, and adoptive cellular immunotherapy, constitutes a novel approach in the management of esophageal cancer (EC). Of noteworthy importance, ICIs have demonstrated efficacy in the management of melanoma and non-small cell lung cancer, and have exhibited encouraging outcomes in the treatment of advanced ESCC (5). An overarching conclusion that the tumor microenvironment (TME) is a multicellular context containing complex stromal-tumor interactions has been well established (6). The induction of proliferation, angiogenesis, inhibition of apoptosis, immune system suppression, and evasion of immune surveillance are intrinsically linked to TME. The tumor cells and surrounding TME cells constantly adapt to the new conditions and promote tumor growth. TME creates a niche for residing and interacting cancer cells with their surrounding endothelial, and immune cells as well as fibroblasts. The reciprocal communication between cancer cells and stromal cells as well as immune cells induces changes in the cellular components of TME, which predisposes cancer cells to metastasis (7, 8). CAFs are a prominent stromal component in the tumor microenvironment (TME) and are present in varying types of solid tumors, making them an important target for treatment (9). Through various mechanisms, activated CAFs can facilitate tumor growth, angiogenesis, invasion, and metastasis, as well as extracellular matrix (ECM) remodeling and even chemoresistance. CAFs communicate with immune cells that infiltrate the tumor microenvironment (TIME), as well as other immunological constituents, by releasing a plethora of cytokines, growth factors, chemokines, exosomes, and other effectual molecules. This phenomenon leads to the molding of an immunosuppressive TIME, which facilitates cancer cells to elude immune surveillance (10). All cellular and non-cellular constituents of the tumor microenvironment can engage in intricate and tightly regulated reciprocal dialogues, thereby promoting cancer initiation, progression, and resistance to therapy. A comprehensive comprehension of the crosstalk between the microenvironment and cancerous cells is indispensable for devising innovative therapeutic strategies (11). CAFs have been identified in divergent types of tumors, including breast cancers and esophageal squamous cancer (12, 13). Accumulating evidence has confirmed that CAFs-specific signatures can be utilized for prognosis prediction in colon cancer ascribing to several markers expressing in CAFs correlated with prognosis (14). Recently, the interplay between CAFs and the tumor immune microenvironment (TIME) has been recognized as a crucial element in driving tumor progression (10). A study has revealed that primary oral squamous cell carcinoma (OSCC) tumors exhibit a negative correlation between WNT2+ cancer-associated fibroblasts (CAFs) and active CD8+ T cells. The use of anti-WNT2 monoclonal antibody has been shown to significantly restore antitumor T-cell responses within tumors and increase active dendritic cells (DCs) in both mouse OSCC and colorectal cancer (CRC) syngeneic tumor models, thereby enhancing the efficacy of anti-PD-1 treatment. Direct interference with CAFs-derived WNT2 has been found to restore DC differentiation and DC-mediated antitumor T-cell responses. Mechanistic analyses have further demonstrated that CAFs-secreted WNT2 suppresses the DC-mediated antitumor T-cell response through the SOCS3/p-JAK2/p-STAT3 signaling cascades. Targeting WNT2 might enhance the ICI efficacy and represent a new anticancer immunotherapy (15). Thus, CAFs were frequently targeted in anti-tumor immunotherapy (16). Yet, the mechanisms by which CAFs regulate the antitumor immune responses in solid tumors are currently not fully understood.

Recent progresses in single-cell sequencing has shed new light on exploring biological systems with revolutionary solutions (17). Different from bulk sequencing, which focuses on averaged data, single-cell sequencing, including transcriptomics, epigenomics, genomics, proteomics and metabolomics sequencing, is a splendid tool to illuminate the cellular and molecular landscape at the single-cell level (18). Single-cell sequencing, besides, has become indispensable in decomposing tissues into cell type or cell states and dissecting the cellular heterogeneity (19). Through single-cell sequencing, conventional dendritic cell (cDC) and distinct macrophage subsets were identified exerting enormous impact on mediating cellular cross-talk in the tumor microenvironment, providing myeloid-targeted immunotherapies for colorectal patients (20). One recent research has offered a novel insight complex cellular architecture and potential therapeutic measures for patients diagnosed with breast cancer (21). Breaking down the complexity of several tumors and characterizing heterogeneous phenotypic states in extraordinary detail (20), single-cell sequencing is quite suitable for ESCC analyses.

Numerous studies have been conducted on CAFs in esophageal squamous cancer (ESCC), but the systematic characteristics of CAFs and their correlation with ESCC prognosis and immunotherapy response are not yet fully comprehended. In this study, we obtained single-cell RNA sequencing (scRNA-seq) data and transcriptome data from accessible databases to differentiate CAF subclusters and establish a CAF-based risk signature for ESCC. We evaluated the clinical significance of the CAF-based signature and further analyzed the immune landscape and responsiveness to immunotherapy associated with it. Finally, we developed a novel nomogram that combines the CAF-based risk signature with clinicopathological features to facilitate the clinical use of CAF features in the prognosis of ESCC. Our findings could provide novel insights into the pathophysiology of ESCC, bringing about personalized treatments and improved outcomes for patients with ESCC.

## 2 Methods

### 2.1 Data collection and processing

ESCC scRNA-seq data was acquired in Gene Expression Omnibus (GEO) database (accession number GSE191756). We screened out single cells with any gene expressed in fewer than three cells or those expressing fewer than 250 genes. The percentage of rRNA and mitochondria was then calculated with the PercentageFeatureSet function in the Seurat R package (22). Consequently, 12118 cells were totally obtained for subsequent analysis.

We further collected transcript data, single-nucleotide variants (SNV), copy number variants (CNV), and corresponding clinical data of ESCC from The Cancer Genome Atlas (TCGA) database. Samples lacking outcome status or survival data were excluded and 94 ESCC samples were obtained, which were utilized for external validation. GSE53624 data with 119 tumor samples and 119 normal ones was used as the training cohort after abandoning samples without follow-up acquired from Gene Expression Omnibus (GEO) database (The clinical characteristics of both the training cohort and the test cohort were exhibited in Supplementary Tables). Based on the literature, ten cancer-associated pathways (Cell Cycle, NRF1, MYC, NOTCH, HIPPO, PI3K, TP53, PI3K, WNT, and TGF-Beta) were identified and analyzed about their gene expression profiles in our dataset.

### 2.2 CAF definition

The scRNA-seq data of ESCC was re-analyzed using the Seurat package, with the aim of providing a systematic characterization of the CAF signature. To start the data preprocessing, cells with less than 250 or more than 6000 expressed genes were removed, and the remaining expressed genes were log-normalized. Next, the FindIntegrationAnchors function was utilized. To reduce the dimensionality of the data, the t-distributed Stochastic Neighbor Embedding (tSNE) method was applied, utilizing a resolution of 0.1 and selecting 30 principal components. The tSNE method employed was non-linear in nature. To classify the single cells into various subgroups, we utilized the FindNeighbors and FindClusters functions (dim = 30 and resolution = 0.1). Additionally, we performed tSNE dimensional reduction using the RuntSNE function. Fibroblasts were annotated according to four marker genes, including PDGFRB, ACTA2, FAP, and NOTCH3. Subsequently, the fibroblasts were re-clustered using the FindClusters and FindNeighbors functions. To define the marker genes for each CAF cluster, we used the FindAllMarkers function with a comparison between different clusters (minpct = 0.35, logFC = 0.5, and adjust p-value < 0.05). We utilized the CopyKAT R package to analyze the CNV characteristics of the CAF clusters and distinguish them from tumor cells and normal ones. Finally, Kyoto Encyclopedia of Genes and Genomes (KEGG) enrichment analysis was conducted on the marker genes using the clusterProfiler package (23).

### 2.3 Identification of hub genes based on CAF

Differentially expressed genes (DEGs) between normal and tumor tissue were identified using the limma package (24, 25), based on criteria of |log2(FoldChange)|>1 and a false discovery rate (FDR)<0.05. Next, the correlations between CAF clusters and DEGs were evaluated, and key CAF-related genes with p<0.01 and cor>0.4 were identified. To identify prognosis-related genes, the survival package was utilized to conduct univariate Cox regression analysis (26). The least absolute shrinkage and selection operator (lasso) was used to reduce the number of genes (27). Multivariate Cox regression analysis was conducted using the stepwise regression method to establish a CAF-based risk signature, which was calculated using the formula: 0.093*ANGPTL7 + 0.15*C6 + 0.121*CSRP1+-0.08*EXPH5 + 0.12*F2RL2 + 0.014*KCNMA1+-0.373*MAGEC3 + 0.143*MAMDC2+-0.188*SLC4A9. The patients were classified into low- and high-risk groups using zero-mean normalization. The predictive value of the risk signature was evaluated using the timeROC package to perform receiver operating characteristic curve (ROC) analysis. The results demonstrated that the risk signature had significant predictive value for patient prognosis. In summary, our analysis provides important insights into the molecular mechanisms underlying tumor development and highlights the potential of CAF-related genes as prognostic biomarkers for cancer patients.

### 2.4 A novel nomogram constructed based on the risk signature

After conducting univariate and multivariate Cox regression analyses based on the risk signature and clinicopathological features (24, 28), a novel nomogram was constructed to predict the prognosis of ESCC using variables with p<0.05 in the multivariate Cox model. The predictive accuracy of the model was evaluated by generating a calibration curve.

### 2.5 Immune landscape analysis

The correlation between the risk signature and the tumor immune microenvironment (TIME) was comprehensively assessed using several algorithms, including CIBERSORT, EPIC, MCPCOUNTER, and TIMER (29). Stromal scores, immune scores, and estimate scores (stromal scores + immune scores) were calculated using the “estimate” R package to evaluate differences in the tumor microenvironment of patients (30, 31). Besides, the proportions of 22 immune cell subtypes were estimated using the CIBERSORT algorithm based on the GSE53624 cohort. The correlation between genes comprising the signature and immune score were further explored to illuminate the great impact those genes exert on immune-related functions.

### 2.6 Response to immunotherapy

Anti-PD-1 or anti-PD-L1 checkpoint inhibition therapy has gained increasing attention as a crucial component of immunotherapy. Transcriptomic data as well as corresponding clinical data from patients who received anti-PD-L1 therapy from the IMvigor210 cohort were collected to evaluate the performance of the risk signature in predicting responsiveness to immunotherapy (immune checkpoint blocks). Additionally, transcriptomic data from the GSE78220 cohort, which included melanoma patients who received anti-PD-1 checkpoint inhibition therapy before treatment, were downloaded.

### 2.7 Consensus clustering analysis and immune infiltration

To further probe into the heterogeneity of ESCC, all ESCC patients were separated into different clusters according to the expression of CAF-related genes with the R package ‘ConsensusClusterPlus’ (22). Differences in survival, TIME, and immune checkpoints were evaluated among subgroups using the same methodology as previously employed. The immune landscape of ESCC patients based on different clusters was demonstrated in the form of heatmap.

### 2.8 RNA isolation and quantitative RT-PCR assay

Total RNA was isolated from ESCC cells or tissues using TRIzol reagent (Thermo Fisher Scientific, Waltham, MA, USA). The complementary DNA (cDNA) was synthesized as per the manufacturer’s instructions, utilizing the RevertAid™ First Strand cDNA Synthesis Kit (Thermo Fisher Scientific). qRT-PCR was performed with SYBR Green PCR kit (Takara Bio, Otsu, Japan) on a StepOne Real-Time PCR system (Thermo Fisher Scientific). The relative gene expression levels were quantified by employing the 2-△△CT method.

### 2.9 Statistical analysis

All statistical analyses were performed using R software (version 4.1.0). The Wilcoxon test was used for comparing two groups, while Spearman or Pearson correlation was used for correlation matrices. Survival differences through K-M curves were assessed using the Log-rank test, where statistical significance was defined as p-value < 0.05.

## 3 Results

### 3.1 CAFs screening based on scRNA-seq samples

**Figure 1:**
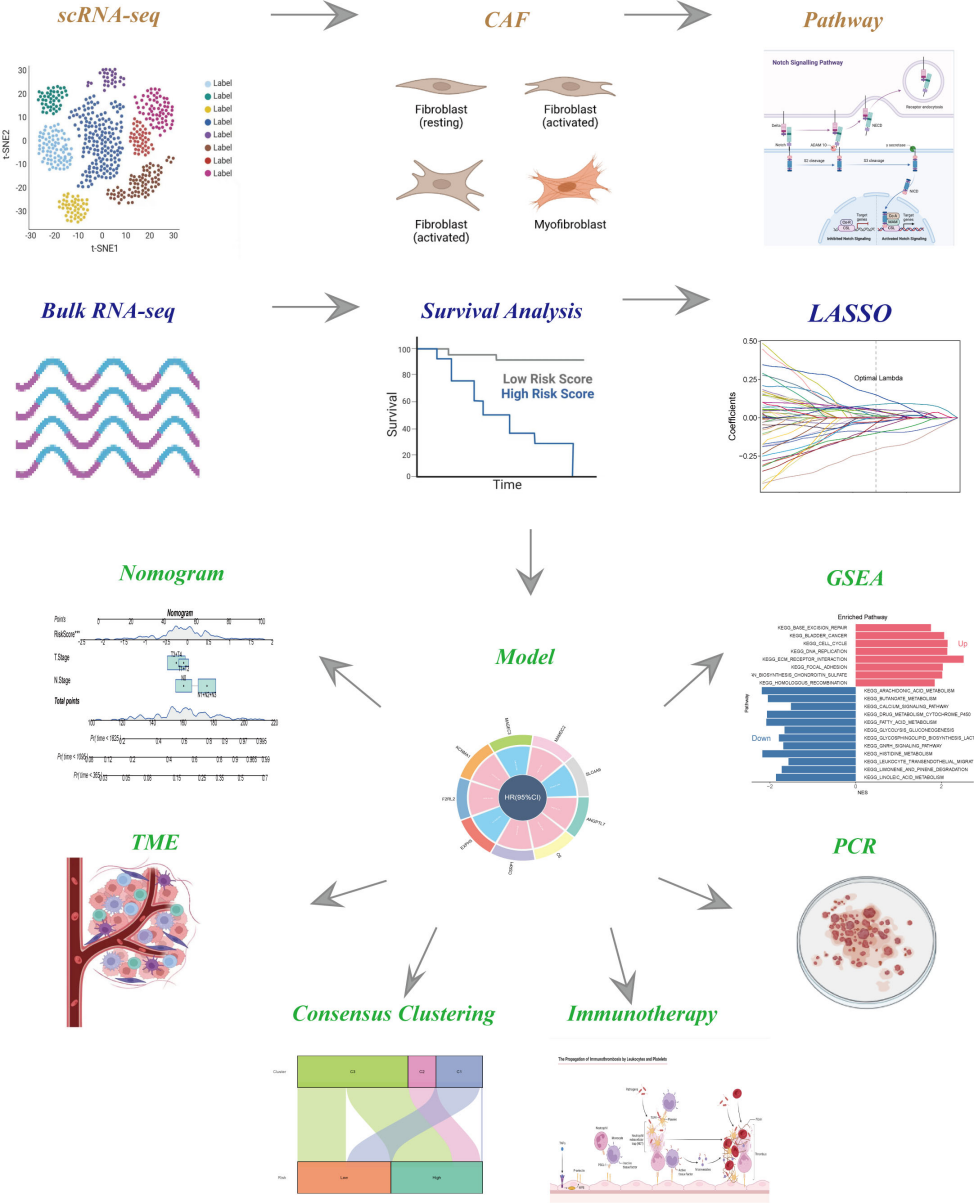
The flow chart of this study.

**Figure 2:**
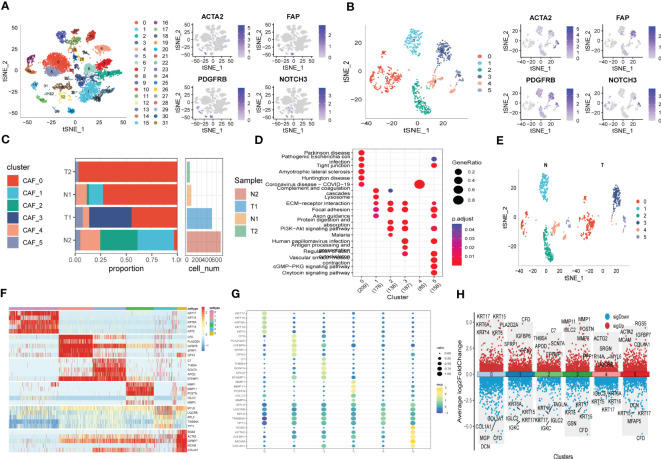
The identification of CAF clusters according to scRNA data of ESCC patients. **(A)** tSNE plots of distribution of 32 clusters and fibroblasts-based marker genes expression. **(B)** tSNE plots of distributions of five fibroblasts after clustering. **(C)** Subgroups in cancer and adjacent tissue and proportion as well as cell number calculation. **(D)** KEGG analysis of five fibroblasts subgroups. **(E)** tSNE distribution of malignant and non-malignant cells predicted by copycat package. **(F)** Heatmap of the top5 marker gene expression of subgroups. **(G)** Bubble diagram of the top5 marker gene expression of subgroups. **(H)** Volcano plot of the top5 marker gene expression of subgroups.

The flow-process diagram of our study was depicted in **Figure 1**. After initial screening, 18024 cells were totally obtained based on the scRNA-seq data. As was shown in **Figure S1**, the details of data preprocessing were demonstrated. After conducting log-normalization and dimensionality reduction, 32 subpopulations were obtained (**Figure 2A**). As presented in **Figure 2B**, six CAF populations were further identified with four marker genes (FDGFRB, FAP, ACTA2, and NOTCH3). The proportions of the six clusters in each cohort were then calculated and the results were illustrated in histograms (**Figure 2C**). Moreover, KEGG analysis illuminated that the DEGs (which were obtained using R package ‘FindVariableFeatures’) were significantly enriched in various pathways, including tight junction, complement and coagulation, focal adhesion and so on (**Figure 2D**). Additionally, distributional differences between tumor and normal cells in the six CAF clusters were presented in **Figure 2E**. In addition, the expression of TOP 5 DEGs were respectively exhibited in heatmap (**Figure 2F**), bubble diagram (**Figure 2G**), and volcano plot (**Figure 2H**).

### 3.2 The exploration of cancer-related pathways in CAF

**Figure 3:**
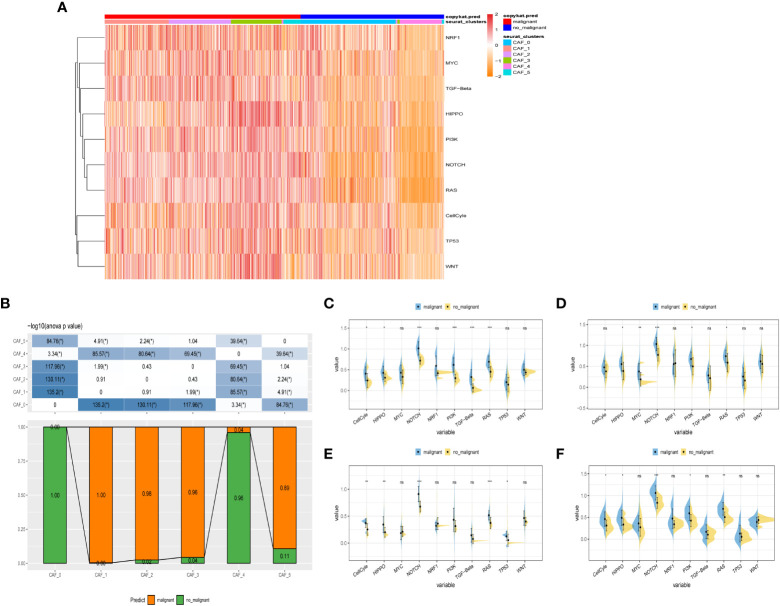
The characteristics of tumor-associated pathways in CAF clusters. **(A)** Heatmap of 10 tumor-associated pathways enriched in CAF cells. **(B)** Comparison between each cluster based on proportions of malignant and non-malignant cells. Comparison of each pathway between malignant and non-malignant cells based on GSVA score in CAF_0 (**Figure S2A**), CAF_1 (**Figure S2B**), CAF_2 **(C)**, CAF_3 **(D)**, CAF_4 **(E)**, CAF_4 **(F)**. (Wilcox. Test, *P < 0.05; **P < 0.01; ***P < 0.001; ns, not significant.).

To probe into the correlations between tumor progression and the CAF clusters, we explored the features of ten tumor-related pathways based on the six CAF clusters. GSVA scores of these divergent pathways were estimated based on various CAF clusters, and the results were depicted in **Figure 3A**. Significant differences about the ratio of malignant cells were obtained in CAF_0 and CAF_4, where the malignant cells accounted for a few proportions. By contrast, the ratio of malignant cells was remarkably higher among CAF_1, CAF_2, CAF_3, and CAF_5 (**Figure 3B**). Besides, slight differences were identified after performing the GSVA analysis of these tumor-related pathways between non-malignant and malignant cells in each CAF cluster (**Figures 3C-F**). (GSVA scores analysis based on CAF_0 CAF_1 was shown in **Figures S2A, B**)

Furthermore, the ssGSEA score of the marker genes (the TOP 5 DEGs obtained in **Figures 2F-H**) were analyzed in each CAF cluster based on the GSE53624 cohort to illustrate the relationships between the CAF clusters and crucial clinicopathological characteristics. Interestingly, tumor samples were found having higher scores compared with normal ones only in CAF_0 and CAF_3, while among the other clusters, normal samples gained significantly higher scores (**Figure S2C**). In addition, ESCC samples of GSE53624 cohort were divided into high-and-low score groups according to the optimal cut-off value with survminer R package. In the CAF_2, CAF_4, and CAF_5 clusters, samples in the low-CAF score subgroup shared a more favorable prognosis compared with those in high-CAF subgroup. CAF_0, CAF_1, and CAF_3, however, were identified not associated with the prognosis of ESCC (**Figure S2D**).

### 3.3 Identification of hub genes correlated with CAF

**Figure 4:**
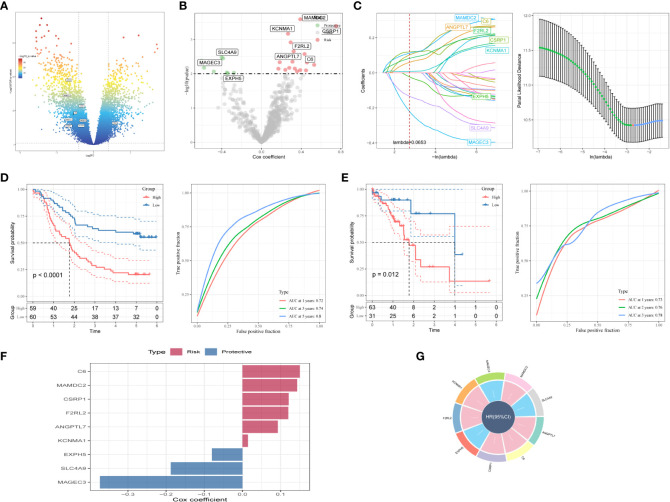
A novel risk signature constructed based on several CAF-related genes. **(A)** Volcano plot of differentially expressed genes between tumor and normal samples in GSE53624 cohort. **(B)** Volcano plot of prognosis-correlated genes obtained by univariate Cox regression analysis. **(C)** Each independent variable’s trajectory and distributions for the lambda. **(D)** K-M and ROC curves of the risk signature in GSE53624 cohort. **(E)** K-M and ROC curves of the risk signature in TCGA cohort. **(F)** The multivariate Cox coefficients for each gene in the risk signature. **(G)** Circle plot showing the multivariate Cox multivariate Cox.

Firstly, DEGs were screened out between normal and tumor samples to establish a risk signature. As depicted in **Figure 4A**, 17080 DEGs were totally obtained, with 7556 down-regulated and 9524 up-regulated DEGs. Among them, a total of 642 genes were identified significantly related with those prognosis-related CAF clusters (**Figure 4B**). After univariate Cox regression analysis, the prognosis of each gene was evaluated, with 8 genes being identified related to protective factors and 18 genes correlated with risk values. Lasso Cox regression analysis was then performed to reduce the number of genes (**Figure 4C**). Furthermore, the stepwise regression method was utilized to develop the risk signature after performing multivariate Cox regression analysis. The signature was composed with nine genes (**Figures 4F, G**), namely complement C6, MAM domain containing 2 (MAMDC2), cysteine- and glycine-rich protein 1 (CSRP1), coagulation factor II thrombin receptor like 2 (F2RL2), angiopoietin like 7 (ANGPTL7), potassium calcium-activated channel subfamily M alpha 1 (KCNMA1), exophilin 5 (EXPH5), solute carrier family 4, sodium bicarbonate cotransporter, member 9 (SLC4A9), and MAGE family member C3 (MAGEC3). And the risk model formula is as follows: RiskScore=“0.093*ANGPTL7 + 0.15*C6 + 0.121*CSRP1+-0.08*EXPH5 + 0.12*F2RL2 + 0.014*KCNMA1+-0.373*MAGEC3 + 0.143*MAMDC2+-0.188*SLC4A9”. The risk score of each sample was calculated using z-mean normalization, and the patients were then separated into high-and-low-risk groups. The Kaplan-Meier survival analysis exhibited that patients in high-risk groups encountered with worse prognosis compared with those in low-risk groups both in GSE53624 (**Figure 4D**) and TCGA cohorts (**Figure 4E**). Additionally, both the GSE53624 and TCGA cohorts exhibited satisfying AUC values of the model, revealing that the predictive power of the signature was excellent. The last, the distribution of patient survival status, risk score, and expression of hub genes in GEO and TCGA cohorts were depicted in **Figure S3**.

### 3.4 Independent risk factors recognition and nomogram construction

**Figure 5:**
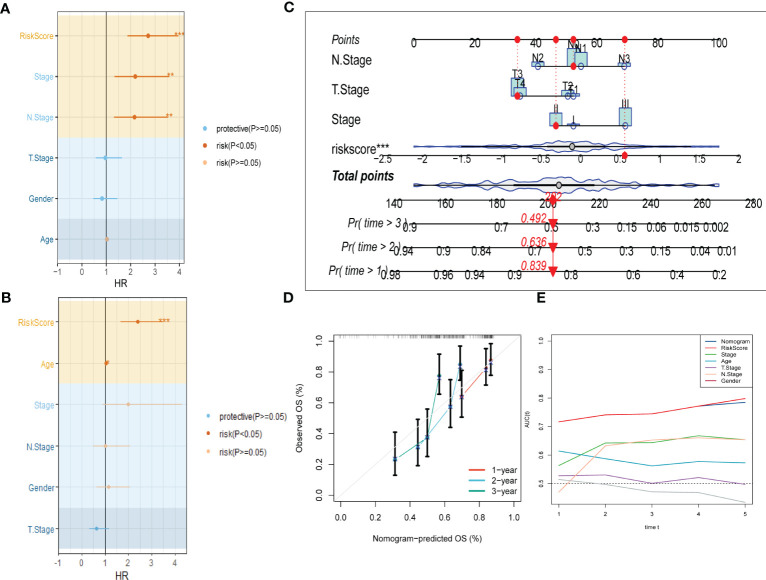
Development of a novel nomogram integrating the risk signature and several clinicopathologic features. **(A)** Results of univariate Cox regression analysis based on risk score and clinicopathologic features. **(B)** Results of multivariate Cox regression analysis based on risk score and clinicopathologic features. **(C)** Construction of the nomogram integrating the T,N-stage clinical stage and risk score. **(D)** Calibration curves for 1, 2, and 3 years of nomogram. **(E)** Evaluation of predictive capacity of nomogram and clinicopathologic features by time-ROC analysis. (*P < 0.05; **P < 0.01; ***P < 0.001).

To improve the accuracy of our predictive model, we incorporated clinicopathological characteristics and the risk score through univariate and multivariate Cox regression analyses. Our multivariate analysis revealed that the risk signature was the most significant independent prognostic factor for ESCC, with a p-value of less than 0.001 (**Figures 5A, B**). We have developed a new nomogram that incorporates T-stage, N-stage, and the risk score (shown in **Figure 5C**). Through calibration plot analysis, this nomogram was found to have strong predictive power for actual survival outcomes (**Figure 5D**). The TimeROC analysis in the TCGA cohort has confirmed that the area under the curve (AUC) of both the nomogram and risk score outperformed other indicators (**Figure 5E**).

### 3.5 Pathway enrichment analysis

**Figure 6:**
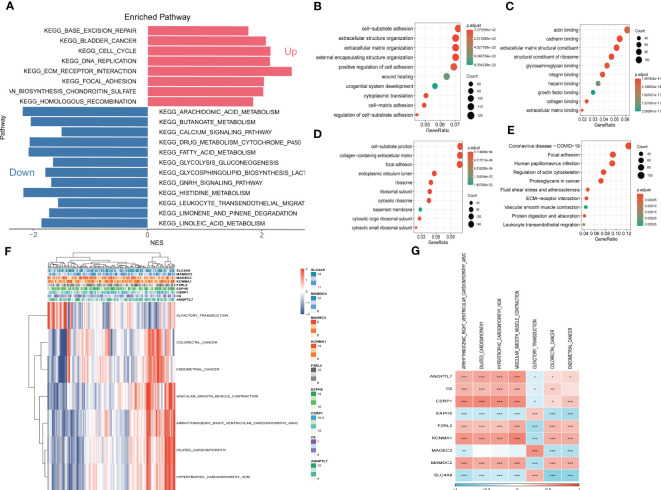
Gene Set Enrichment Analysis (GSEA) **(A)** Gene Set Enrichment Analysis of up-regulated and down-regulated genes **(B) **GO-BP analysis **(C) **GO-CC analysis **(D) **GO-MF **(E) **KEGG analysis **(F) **Heatmap exhibiting enrichment score for key pathways based on the hub genes. **(G)** Gene-pathway correlation heatmap.

To explore the fundamental functions those DEGs play in initiation and progression of ESCC, Kyoto Encyclopedia of Genes and Genomes (KEGG) and Gene Ontology (GO) analysis were conducted. As shown in **Figures 6A–E**, the up-regulated genes were most enriched in base excision repair, cell cycle, and DNA replication, while the down-regulated genes were significantly correlated with arachidonic acid metabolism, calcium signaling pathway, and histidine metabolism. Likewise, the results of GO analysis were presented in **Figures 6B-D**. Furthermore, Gene Set Enrichment Analysis was conducted based on the nine genes involved in the risk signature. The results illustrated that 7 pathways were remarkably associated with these nine genes (**Figures 6F, G**). Interestingly, olfactory transduction was positively correlated with the genes except for EXPH5, MAGEC3, and SLC4A9, which were identified to have protective values in ESCC, indicating that olfactory transduction might suppress the immigration and progression of ESCC.

### 3.6 Immune infiltrations landscape and relationship between risk genes and immunity

**Figure 7:**
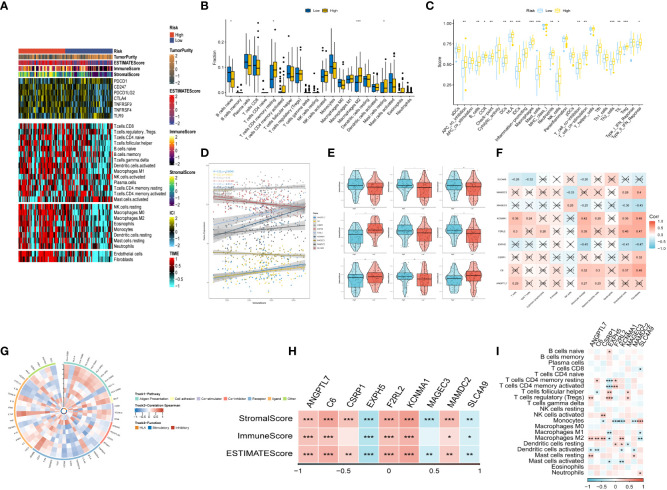
The immune infiltrations analysis **(A)** Heatmap of results on immune cells of tumor microenvironment (TME) in ESCC with multialgorithm, including existing data from platform TIMER and MCP-counter. TME-related scores were exhibited in the top bar. **(B)** Comparison of proportions of 22 immune-related cells between high-and-low-risk groups. **(C)** Comparison of proportions of immune-related functions between high-and-low-risk groups. **(D, E)** Correlations between the nine hub genes and immune score. **(F, I)** Correlations between nine hub genes and 22 immune-related cells. **(G)** The correlation analysis between nine hub genes and 75 immune-associated genes. **(H)** Correlations between the four nine genes and immune score, stromal score, estimate score. (*p < 0.05, **p < 0.01, ***p < 0.001).

After conducting an investigation into the landscape of immune and stromal cell infiltrations in both low- and high-risk groups, **Figure 7A** demonstrated that patients in the high-risk group exhibit higher proportions of immune and stromal cell infiltrations when compared to those in the low-risk group. Besides, the immune cells proportions between the low-and-high-risk groups were estimated using the CIBERSORT algorithm. It was found that the high-risk group had higher proportions of resting memory CD4 T cells, Macrophages (M2), and resting mast cells, while naive B cells were more enriched in low-risk group (**Figure 7B**). **Figure 7C** exhibited the results of immune-related functions differences between high-and-low-risk groups.

Additionally, the relationship between risk genes and immunity was probed into. On the one hand, **Figures 7D–H** demonstrated that the protective genes (including EXPH5, MAGEC3, and SLC4A9) were found negatively correlated with stromal score, immune score, and estimate score. On the other hand, the risk genes (including PR1, F2RL2, KCNMA1, and MAMDC2) were identified positively correlated with divergent immune cells (**Figures 7F, I**). Finally, the reciprocal communication between the 75 immune-related genes and the nine model genes were displayed in **Figure 7G**.

### 3.7 Response prediction of risk signature to immunotherapy

**Figure 8:**
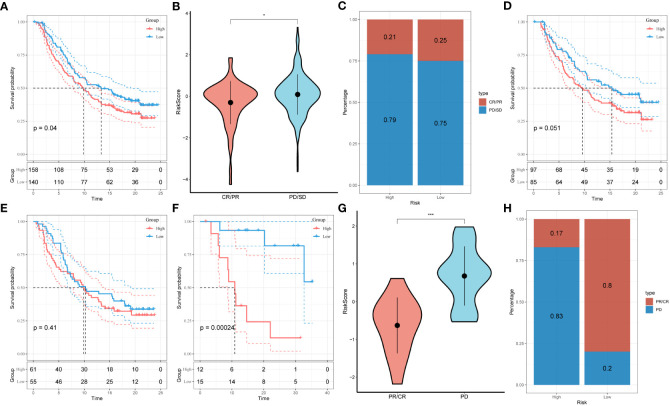
Prediction of responsiveness to immunotherapy using our signature based on public database. **(A)** Prognostic differences between risk subgroups in the IMvigor210 cohort. **(B)** Differences among immunotherapy responses based on risk scores in the IMvigor210 cohort. **(C)** Distribution of immunotherapy responses based on risk subgroups in the IMvigor210 cohort. **(D)** Prognostic differences between risk subgroups based on early stage (stage I-II) in the IMvigor210 cohort. **(E)** Prognostic differences between risk subgroups based on advanced patients (stage III-IV) in the IMvigor210 cohort. **(F)** Prognostic differences between risk subgroups in the GSE78220 cohort. **(G)** Differences among immunotherapy responses based on risk scores in the GSE78220 cohort. **(H)** Distribution of immunotherapy responses based on risk subgroups in the GSE78220 cohort.

Under the circumstances that T-cell immunotherapy has gained great achievements in recent years, we performed the assessment of prognostic value of our signature for immune-checkpoint therapy in GSE78220 and IMvigor210 cohorts. Divergent degrees of responsiveness of anti-PD-L1 receptor blockers were identified in the 348 patients from the IMvigor210 cohort, including partial response (PR), complete response (CR), progressive disease (PD), and stable disease (SD). As depicted in **Figures 8A-C**, patients in the high-risk group accounted for more proportions in PD/SD, and had worse prognosis than those in the low-risk group. Besides, SD/PD patients tended to gain higher risk scores than CR/PR patients. However, significant survival differences were identified neither in Stage I+II nor in Stage III+IV patients between the different risk subgroups (**Figures 8D, E**). To validate our findings, the GSE78220 cohort was enrolled for further analysis. Corresponding with the results from IMvigor210, patients who achieved partial or complete response had lower risk scores and were less likely to be in the high-risk group (**Figures 8F-H**).

### 3.8 Consensus clustering and immune infiltrations

**Figure 9:**
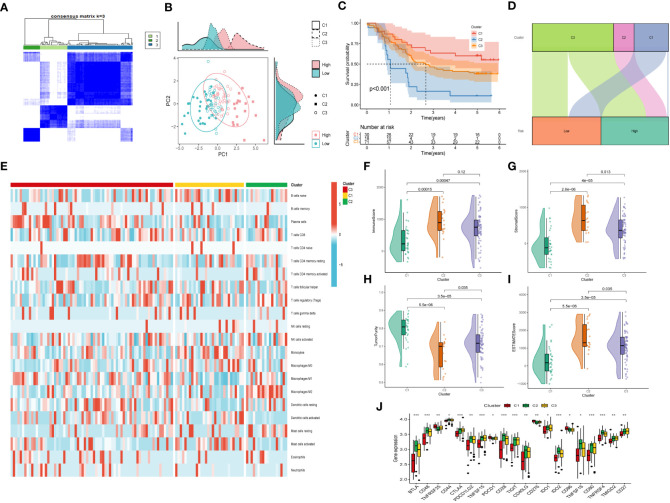
Consensus Clustering based on nine prognostic CAF-related genes expression. **(A)** ESCC patients were divided into three clusters (k=3). **(B)** PCA depicted the distribution for clusters. **(C)** Survival analysis based on the three clusters. **(D)** The Sankey diagram of the connection between clusters and high-and low-risk group. **(E)** Immune infiltrations based on three clusters. **(F)** ImmuneScore difference between three clusters. **(G)** SromalScore difference between three clusters. **(H)** TumorPurity difference between three clusters. **(I)** ESTIMATEScore difference between three clusters. **(J)** Expression difference of immune checkpoints between three clusters. (*p < 0.05, ***p < 0.001).

Moreover, unsupervised consensus clustering was conducted to explore molecular subtypes based on the expression of CAF-related genes comprising the risk signature. With k = 3 deemed as the optimal clustering stability, patients in GSE53624 cohort were grouped into three clusters (**Figure 9A**). The ridge plot exhibited the distribution of various clusters (**Figure 9B**). Besides, as presented in Sankey diagram (**Figure 9D**), Cluster 1(C1) and Cluster 3(C3) made up the low-risk group while the high-risk group was comprised of Cluster 2 (C2) and Cluster 3 (C3). Subsequent survival analysis illustrated those patients in the C1 group had the most favorable prognosis, while patients in the C3 group had the worst clinical outcomes (**Figure 9C**). The immune landscape based on different clusters were shown in heatmap (**Figure 9E**), which indicated that C2 cluster beard the highest immune cell infiltrations. The TME scores of varying clusters were then calculated, revealing that C2 cluster had the highest immune, stromal, and estimate score as well as lower tumor purity than the other clusters. (**Figures 9F-I**). After applying the immune checkpoints inhibitors analysis, it was identified that C2 cluster was significantly correlated with BTLA, CTLA4, CD48, and so on, suggesting that patients involved in the C2 group might benefit from immunotherapy, especially anti-PD-1 receptor blockers.

### 3.9 Drugs sensitivity

**Figure 10:**
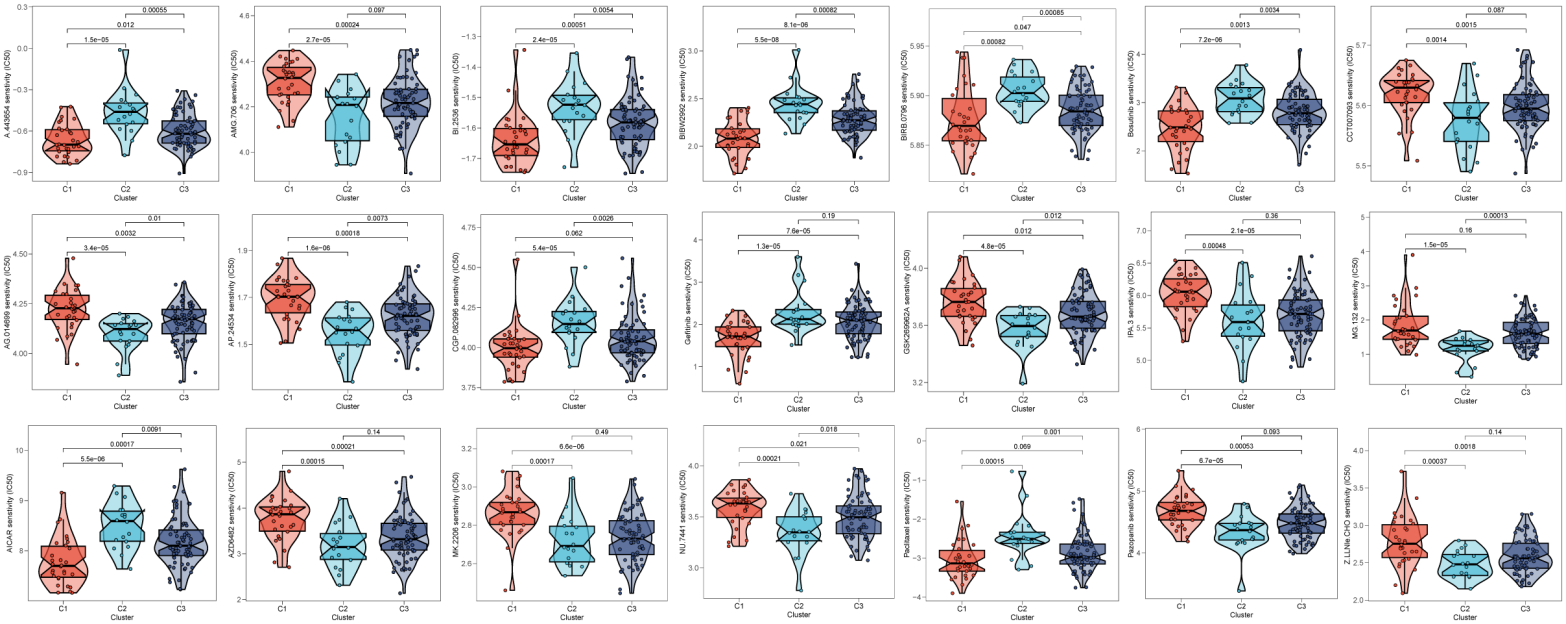
Prediction of chemotherapy drug sensitivity in ESCC patients based on different clusters. The experiment of ESCC risk-related genes. (*p < 0.05, **p < 0.01, ***p < 0.001).

After comparing the efficacy of various chemotherapeutic agents across different clusters, we discovered that patients belonging to cluster 2 (C2) exhibited elevated IC50 values for chemotherapeutic medications such as Bosutinib, Gefitinib, and AICAR. Additionally, patients in cluster 1 (C1) were observed to be more receptive to AMG.706, IPA.3, and the like, while those in cluster 3 (C3) demonstrated poorer response rates to the majority of chemotherapeutic treatments (**Figure 10**).

### 3.10 The experiment of genes involved in the risk signature

**Figure 11:**
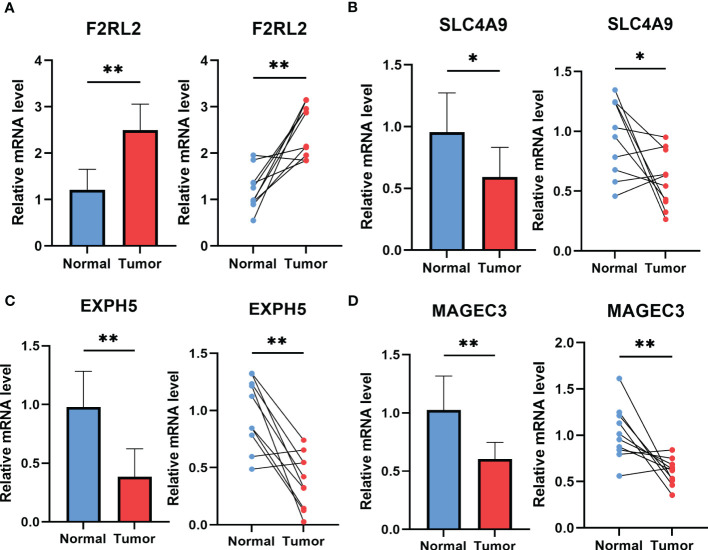
The expression of F2RL2 **(A)**, SLC4A9 **(B)**, EXPH5 **(C)** and MAGEC3 **(D)** in normal esophageal tissue and ESCC tissue of patients. t-test was used to compare the expression of genes between normal and tumor. *p < 0.05, **p < 0.01, ***p < 0.001.

To explore potential ESCC cancer risk-related genes, four genes involved in the risk signature were selected for further validation in ESCC patients. As demonstrated in **Figure 11**, F2RL2 exhibited elevated expression levels in tumors, whereas SLC4A9, EXPH5, and MAGEC3 exhibited significantly reduced expression levels in tumors. These distinctions align with our bioinformatic findings, suggesting that these genes may serve as innovative biomarkers for early ESCC diagnosis.

## 4 Discussion

The tumor microenvironment, as well known, encompasses the non-cancerous cells and components that are found within a tumor, along with the molecules that they produce and release (32).The continuous interactions between tumor cells and the tumor microenvironment are crucial in determining the tumor’s initiation, progression, metastasis, and response to therapies (33). Numerous studies have provided compelling evidence to support the idea that there is a dynamic crosstalk between tumor cells and stromal cells, which plays a critical role in tumor progression (34). By understanding the mechanism of this interaction, there is an opportunity to develop enhanced therapeutics that target multiple components of the TME simultaneously, ultimately increasing the likelihood of favorable patient outcomes (35). Considering that cancer-associated fibroblasts (CAFs) have been identified linked with tumor initiation and progression (36), a comprehensive exploration on characterization and classification of CAFs of ESCC via scRNA-seq data was performed. Six distinctive CAF clusters were identified, which might exert enormous influence on divergent biological regulation of the TME. Accumulating evidence has confirmed that CAF-related gene signature has great prognostic value in ESCC (37, 38). Correspondingly, three clusters in our data were found significantly associated with ESCC prognosis. After analyzing the tumor-related pathways based on the CAF clusters, HIPPO, NOTCH, and RAS were identified significantly enriched in malignant parts in CAF_2, CAF_3, CAF_4, and CAF_5 clusters. It has been revealed that HIPPO and RAS signaling pathways can impel the tumor proliferation and immigration in ESCC (39, 40). A recent study has illuminated that via Notch signaling pathway METTL3-mediated m6A mRNA modification can propel esophageal cancer initiation and progression (41). Besides, it has been discovered that a depletion of PARK2 promotes the progression of esophageal squamous cell carcinoma (ESCC) via the Hippo/YAP axis, whereas overexpression of PARK2 suppresses tumor progression of ESCC through the Hippo signaling pathway. Consequently, as a newfound regulator of Hippo signaling, the manipulation of PARK2 activity or gene expression levels may prove to be a promising strategy for treating esophageal cancer (39). According to the genes included in the CAF clusters which were identified significantly correlated with ESCC prognosis, a novel risk signature based in CAFs were established. Our model comprised of 6 risk genes (C6, MAMDC2, CSRP1, F2RL2, ANGPTL7, and KCNMA1), and 3 protective genes (EXPH5, SLC4A9, and MAGEC3). It has been revealed that ANGPTL7 had an excellent performance as a surrogate marker of microvascular invasion on hepatocellular carcinoma (42). Besides, several genes (including MAMDC2, F2RL2, and KCNMA1) were enrolled as biomarker for the prognosis of varying cancers (43–45). It has been confirmed that MAGEC3 can stimulate cancer metastasis via intriguing epithelial-mesenchymal and immunosuppression in ESCC (46). The GSEA analysis was then applied, demonstrating that protective genes were enriched in olfactory transduction, while risk genes were remarkably associated with other pathways, such as vascular smooth muscle contraction, dilated cardiomyopathy, colorectal cancer and so on. Interestingly, PLK1 has been confirmed suitable for cancer therapy due to its function in regulating contraction of postmitotic smooth muscle cells (47). Moreover, F2RL2 was identified significantly correlated with initiation and progression of colorectal cancer (48). Several researches suggested that high burden of doxorubicin can threaten cancer patients’ health owing to dilated cardiomyopathy (49). Furthermore, the novel signature was confirmed to have excellent prognostic value in ESCC after applying TCGA ESCC cohort for external validation. After categorizing the patients into high- and low-risk groups based on the median risk score, the subsequent analysis revealed that the low-risk group had a significantly better prognosis than the high-risk group. Additionally, both univariate and multivariate Cox regression analyses verified that the risk score was an independent predictor of overall survival (OS). A nomogram was constructed based on the risk signature, which displayed a high degree of consistency between the predicted and observed results for the OS of ESCC patients. Consequently, the study’s findings illustrated that the risk signature created was a dependable tool for accurately predicting the prognosis of ESCC patients. With the risk signature, earlier diagnosis and therapy can be received in ESCC patients. The identified CAF-related gene signature provides a potential prognostic tool for predicting patient outcomes and may help guide treatment decisions for ESCC patients. The signature has the potential to improve patient stratification and identify those who may benefit from more aggressive treatment strategies or targeted therapies. In addition, the identification of the specific genes in the CAF signature provides potential targets for therapeutic intervention, such as drugs that target the overexpressed risk genes or enhance the expression of the protective genes.

The vigorous development of cancer immunotherapy has shed a novel light on the cancer treatment, which extremely depended on the comprehensive perception of immune landscape in tumor microenvironment (50). The tumor microenvironment is a complex ecosystem comprised of diverse cell types that significantly impact cancer biology and the effectiveness of therapeutic interventions (51). Considering that such a bunch of ESCC patients still suffer from the unfavorable prognosis in spite of receiving immunotherapy ascribing to immune escape or immune tolerance (52), we explored the immune landscape of ESCC based on the CAF-related risk signature. It was found that the high-risk group had a higher proportion of immune cells infiltration. Nevertheless, macrophages (M2) were identified significantly enriched in high-risk group in the subsequent analysis, which has been confirmed to incite immune tolerance in cancer immunotherapy (53). Taken above results into consideration, we infer that patients in low-risk group are more likely to benefit from immunotherapy. The relationship between immune infiltration and genes composing the risk signature were further analyzed. Risk genes were positively correlated with immune score while protective genes were negatively associated with immune score. In addition, to probe into the response to anti-cancer immunotherapy, IMvigor210 and GSE78220 cohorts were enrolled for analysis. Within the two cohorts, patients belonging to low-risk group account for higher proportions of partial response (PR) and complete response (CR) after immunotherapy of anti-PD-L1 receptor blockers. Consistent with above results, patients in low-risk group benefit more from immunotherapy than those in high-risk group. However, immunotherapy in ESCC is far from anti-PD-1 or anti-PD-L1, further researches are urgently needed to provide precise and comprehensive management for patients diagnosed with ESCC.

An overarching conclusion that esophageal squamous cancer is highly heterogeneous has been well received. Uncovering the heterogeneity of ESCC could revolutionized the management of this malignant cancer and provide patients with more favorable prognosis (51). Thus, we preformed consensus clustering in GSE53624 cohort based on the risk signature. Cluster 2(C2) was found made up by the high-risk group and with the worst prognosis. Besides, C2 had the highest immune score, stromal score, and estimate score and was identified significantly correlated with several immune checkpoints (including BTLA, CD48, CD44, CTLA4, CD28, IDO2, and so on), revealing that patients in Cluster 2 might be suitable for immunotherapy of immune checkpoints inhibitors. Last but not the least, various CAF-associated genes implicated in the risk signature were subsequently subjected to validation using ESCC tissues. In line with our bioinformatic findings, F2RL2 was determined to be highly expressed in tumors; conversely, SLC4A9, EXPH5, and MAGEC3 were observed to be significantly under-expressed in tumors. It has been revealed that F2RL2 can promote the tumorigenesis and immigration of breast cancer (44), indicating that these genes may serve as innovative biomarkers for early ESCC diagnosis. Although our study provides valuable insights, there are some limitations that require attention. Firstly, our risk signature was established based on retrospective data obtained from public databases. Thus, more prospective and multi-center cohorts of ESCC are necessary to mitigate any potential bias. Secondly, our risk signature only predicts the responsiveness to anti-PD-L1 immunotherapy, further research is urgently needed to assess its potential for predicting the response to other precision therapies in the future.

## 5 Conclusion

In our study, we conducted an extensive investigation into the populations of CAFs in ESCC and identified six distinct CAF clusters. Three of these clusters were significantly associated with ESCC prognosis and were used to establish a prognostic risk signature consisting of nine genes based on the CAFs. Moreover, we developed a novel nomogram that combined the risk signature with clinicopathological characteristics, which exhibited excellent performance in predicting the clinical outcomes of patients with ESCC. We also observed that our risk signature was associated with tumor mutations and immune landscape, and that it is suitable for predicting the responsiveness of ESCC patients to immunotherapy targeting PD-L1 blockade.

## Data availability statement

The datasets presented in this study can be found in online repositories. The names of the repository/repositories and accession number(s) can be found within the article/**Supplementary Materials**.

## Ethics statement

All human experiments in this study have been approved by the Ethics Committee of the First Affiliated Hospital of Nanjing Medical University. All subjects gave their informed consent for inclusion before they participated in the study. The study was conducted in accordance with the Declaration of Helsinki, and approved by the Ethics Committee of the First Affiliated Hospital of Nanjing Medical University (protocol code No.2019-SRFA-005; 27 February 2019).

## Author contributions

QR, PZ, HL and YY contributed conception and design of the study. XZ finished the dada collection. XZ and YF performed the statistical analysis. QR wrote the first draft of the manuscript. HL and YY revised the manuscript. LL, HL and YY gave the final approval of the version to be submitted. All authors contributed to the article and approved the submitted version.
