# Supplementary material for: A fibroblast-associated signature predicts prognosis and immunotherapy in esophageal squamous cell cancer
Source: Front Immunol. 2023 May 29;14:1199040. doi: 10.3389/fimmu.2023.1199040 (PMC10258351; doi:10.3389/fimmu.2023.1199040)
Supplement: Supplementary file 1 [file DataSheet_1.docx]

**Supplementary Material**

**Supplementary Table S1** The clinical characteristics of both the training cohort and the test cohort.

| **Variables** | **Group** | **Training set(GSE53624)** | **Testing set (TCGA)** |
| --- | --- | --- | --- |
| **Age** | **<=65** | 88 | - |
|  | **>65** | 31 | - |
| **Gender** | **Female** | 21 | 15 |
|  | **Male** | 98 | 80 |
| **Vital status** | **Alive** | 46 | 64 |
|  | **Dead** | 73 | 31 |
| **Survival time** |  | 1111.832 | 458.505 |
| **Clinical Stage** | **I** | 6 | 7 |
|  | **II** | 47 | 56 |
|  | **III** | 66 | 26 |
|  | **IV** | 0 | 4 |
|  | **unknow** | 0 | 2 |
| **T stage** | **T1** | 8 | 8 |
|  | **T2** | 20 | 32 |
|  | **T3** | 62 | 49 |
|  | **T4** | 19 | 4 |
|  | **Tx** | 0 | 2 |
| **N stage** | **N0** | 54 | 55 |
|  | **N1** | 42 | 28 |
|  | **N2** | 13 | 6 |
|  | **N3** | 10 | 3 |
|  | **Nx** | 0 | 3 |
|  |  |  |  |

<https://www.jianguoyun.com/p/DQZ_tyAQjdemCxie4f4EIAA>
