# Supplementary figures and images for: A fibroblast-associated signature predicts prognosis and immunotherapy in esophageal squamous cell cancer
Source: Front Immunol. 2023 May 29;14:1199040. doi: 10.3389/fimmu.2023.1199040 (PMC10258351; doi:10.3389/fimmu.2023.1199040)

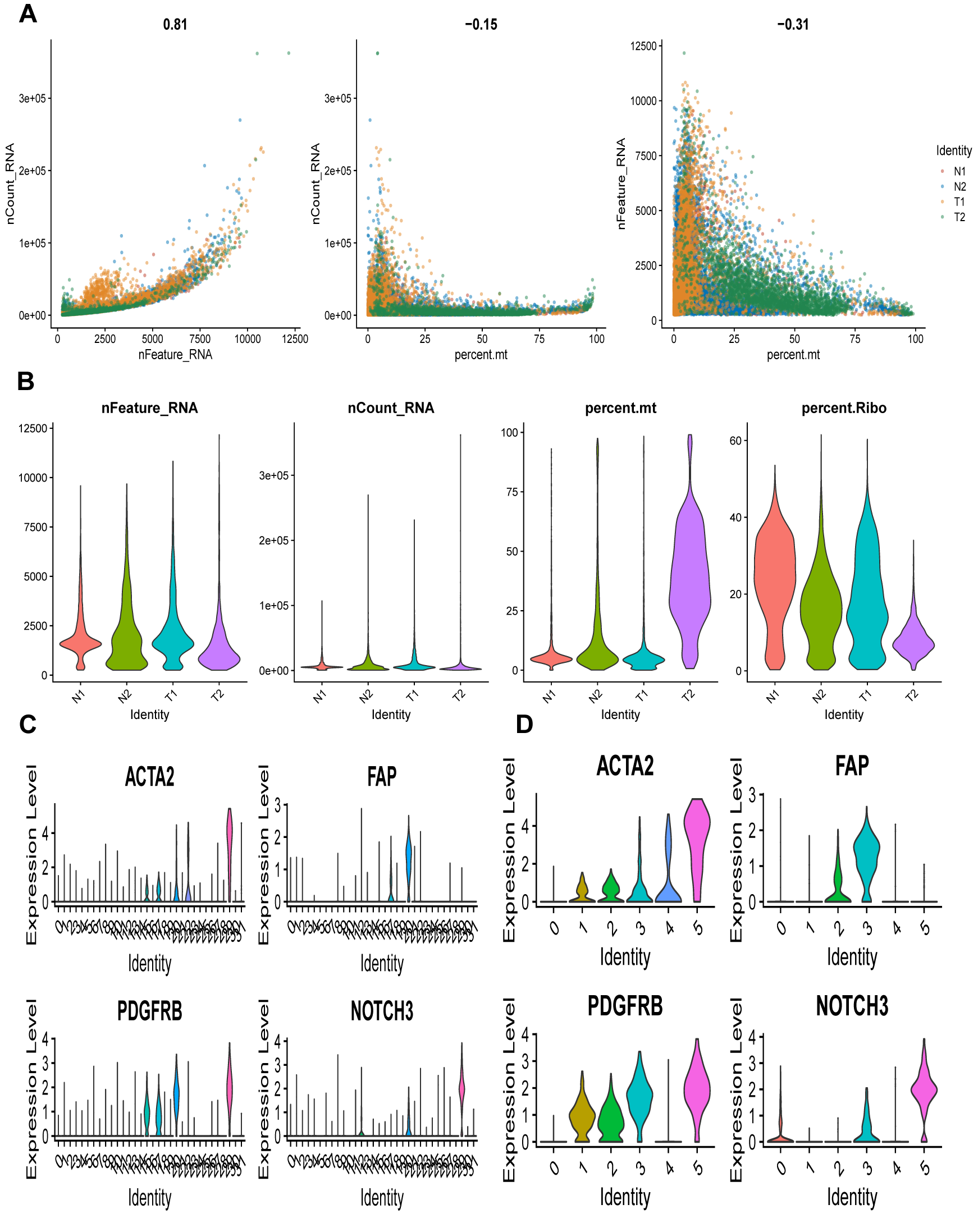

Supplement: Supplementary file 2 [file Image_1.tif]

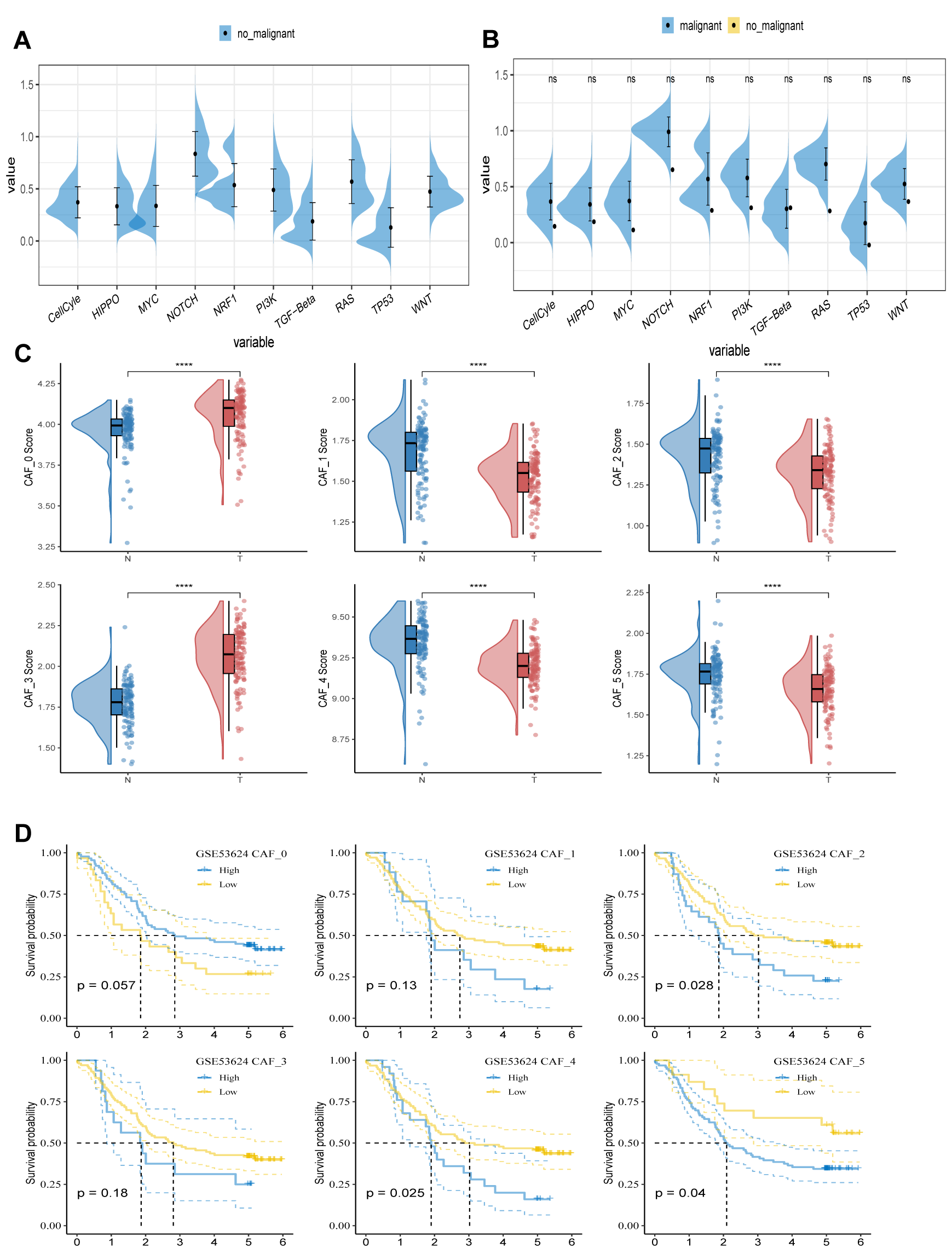

Supplement: Supplementary file 3 [file Image_2.tif]

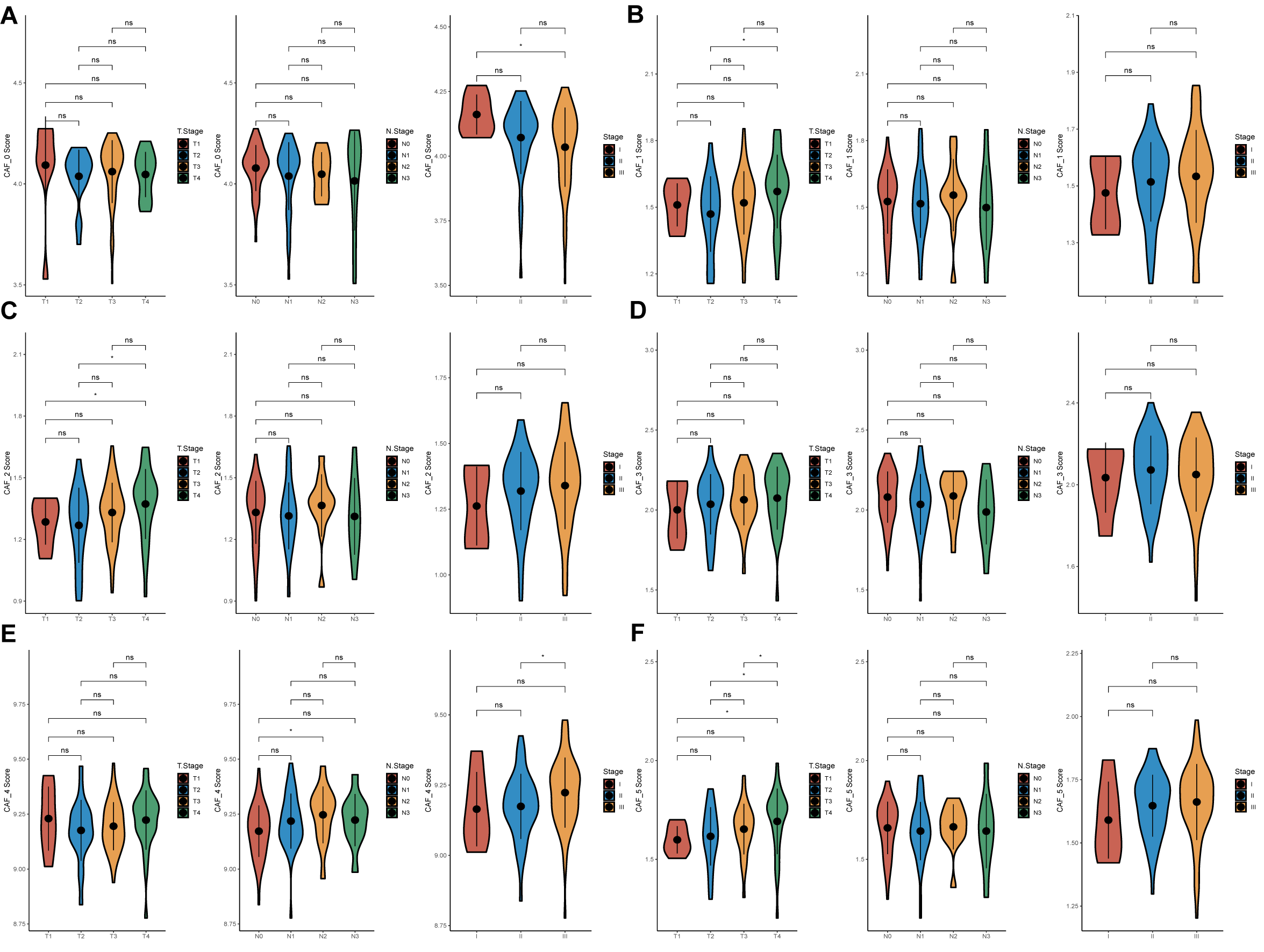

Supplement: Supplementary file 4 [file Image_3.tif]

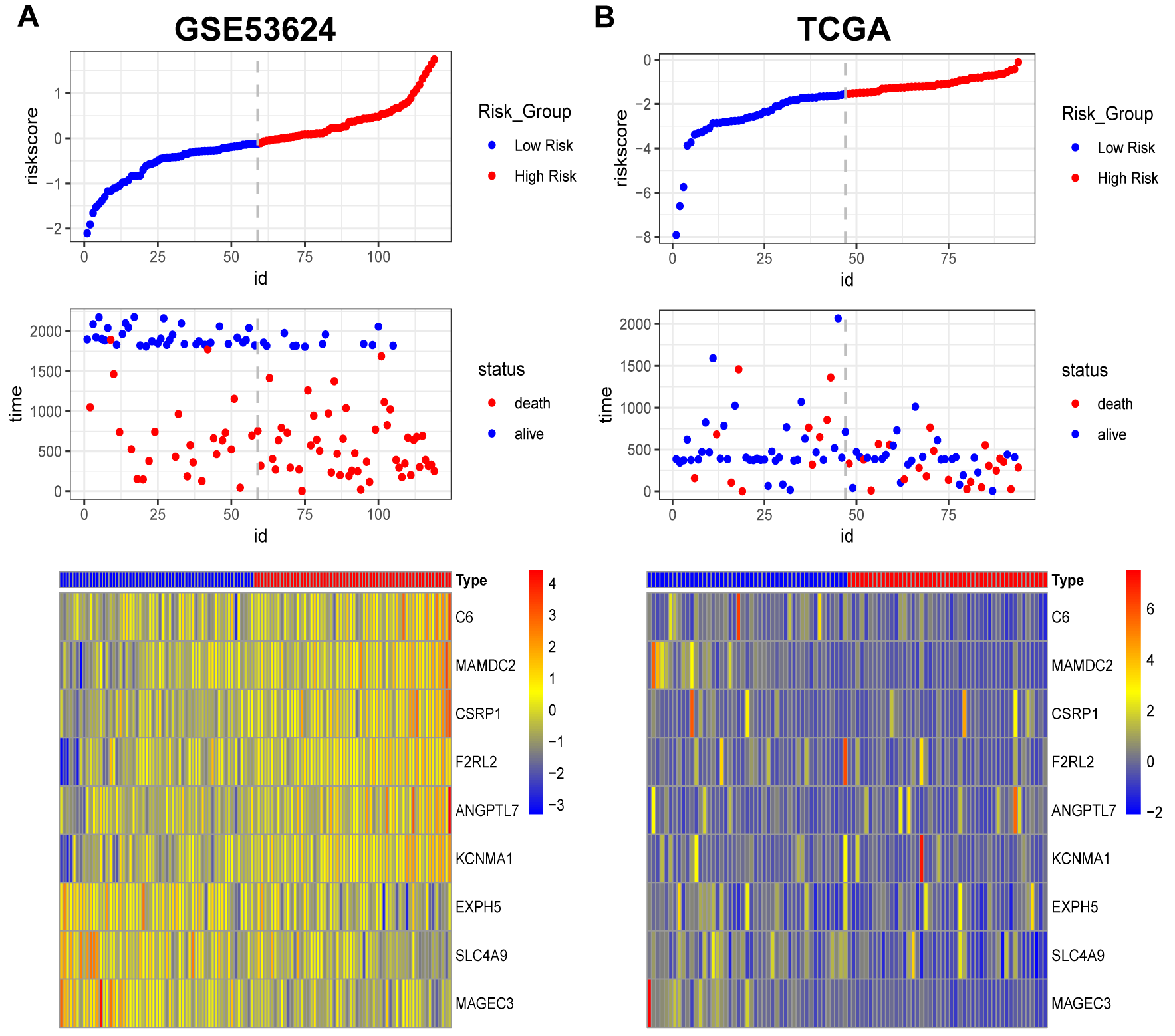

Supplement: Supplementary file 5 [file Image_4.tif]
